# Vocal fold fixation due to proximal stenosis progression complicating idiopathic subglottic stenosis

**DOI:** 10.1007/s00405-019-05494-6

**Published:** 2019-06-11

**Authors:** S. A. R. Nouraei, E. B. Dorman, J. Johnston, D. E. Vokes

**Affiliations:** 1 Department of Ear Nose and Throat Surgery, The Robert White Centre for Airway Voice and Swallowing, Poole Hospital NHS Foundation Trust, Poole, UK; 2 Department of Otorhinolaryngology-Head and Neck Surgery, Auckland City Hospital, Auckland, New Zealand

**Keywords:** Idiopathic subglottic stenosis, Laryngotracheal stenosis, Proximal stenosis progression

## Abstract

**Purpose:**

This study examined the incidence and risk factors for vocal fold fixation due to proximal progression of idiopathic subglottic stenosis (ISS) over the course of serial treatments.

**Methods:**

Records of 22 consecutive patients with ISS treated between 2004 and 2016 were retrospectively reviewed. Patient, stenosis, and treatment details were recorded. Cox regression was used to identify independent predictors of vocal fold fixation.

**Results:**

All patients were female and mean age at diagnosis was 46 ± 7 years. In five patients, the stenosis was within 15 mm of the glottis at first treatment. Vocal fold fixation due to proximal stenosis progression occurred in seven (32%) patients. It led to permanent hoarseness due to unilateral vocal fold fixation in two patients and caused airway compromise due to bilateral vocal fixation in two other patients. No airway-related deaths occurred and no patient required a tracheostomy. Stenosis incision using coblation or potassium titanyl phosphate laser, and an initial glottis-to-stenosis (GtS) distance < 15 mm significantly increased the risk of proximal stenosis progression on univariable analysis.

**Conclusion:**

Vocal fold fixation due to proximal stenosis progression is a significant complication of idiopathic subglottic stenosis and causes permanent voice and/or airway sequelae. It should be actively looked for and documented every time a patient is assessed. If a reduction in the GtS distance is observed, definitive surgery should be promptly considered before proximal stenosis progression compromises the efficacy and safety of definitive treatment or, worse, causes vocal fold fixation.

## Introduction

Idiopathic subglottic stenosis (ISS) is a progressive fibromatosis of the lamina propria of the subglottis and proximal cervical trachea [1]. It occurs exclusively in women who are mainly of North European ancestry [2] and has a population incidence of two per million [3]. ISS presents with exertional dyspnoea, effort intolerance and chronic stridor, most frequently during the fourth and fifth decades of life [4]. Therapeutic approaches to managing this condition can be broadly divided into those that aim to cure it by preventing recurrent fibrosis, and those that aim to maintain an adequate airway whilst accepting the condition’s underlying propensity for progressive fibrosis causing periodic recurrences of the stenosis.

Curative approaches to ISS recognise the importance of the overlying epithelium as the long-term modulator of wound healing and an as-yet uncharacterised but likely hormone-mediated [5] intrinsic abnormality of this tissue as the cause of the condition [6]. These approaches involve removing the fibrosis-forming subglottic mucosa and replacing it with healthy epithelial tissue. The same conceptual approach of replacing disease-forming epithelium with normal autologous tissue to prevent recurrent fibrosis has been used in other fibromatoses like Dupuytren’s disease [7, 8] and plantar fibromatosis [9]. Mucosal replacement to achieve ‘biological inhibition’ of fibrosis is accomplished either with vascularised tracheal mucosa in cricotracheal resection [10] or by facilitating implantation and incorporation of cutaneous or buccal epithelium within the subglottic airway by wrapping a graft of tissue around a temporary airway stent as part of either an open laryngotracheal reconstruction [4] or an endoscopic laryngotracheoplasty [1] with biological inhibition.

Maintenance approaches to managing ISS recognise the morbidity that could accompany definitive approaches and accept the need for multiple, but less invasive procedures to maintain an adequate airway. This is accomplished by incising and dilating the stenosis and by pharmacologically modulating the scar response [11] to maximise intervention-free intervals [12]. More recently, serial intralesional injections of corticosteroids [13, 14] and systemic administration of antimetabolites [15] have been used as adjunctive or single-modality airway maintenance treatments.

Some 20–40% of patients with ISS achieve long-term disease remission following a single endoscopic treatment [3, 16] and as such, an initial endoscopic approach is appropriate for patients with de novo disease. A significant proportion of patients who experience recurrences of ISS still prefer infrequent minimally invasive treatments over definitive treatments that carry greater risks of complications and permanent morbidity. Specifically, cricotracheal resection places the recurrent laryngeal nerves and the cricothyroid muscles and joint at risk, which may cause permanent voice change [17]. There is also a risk of stenosis recurrence in the long term [18] which appears to be associated with the extent of the resection that needs to be performed. Conversely, implanting distant epithelial cells within the airway frequently causes chronic problems with cough and mucus, which, for some patients, effectively replaces one chronic condition (recurrent stenosis) for another (chronic cough, and mucus retention and plugging). As such, the decision on whether to continue with airway maintenance treatment or to opt for a curative treatment can be nuanced.

**Fig. 1:**
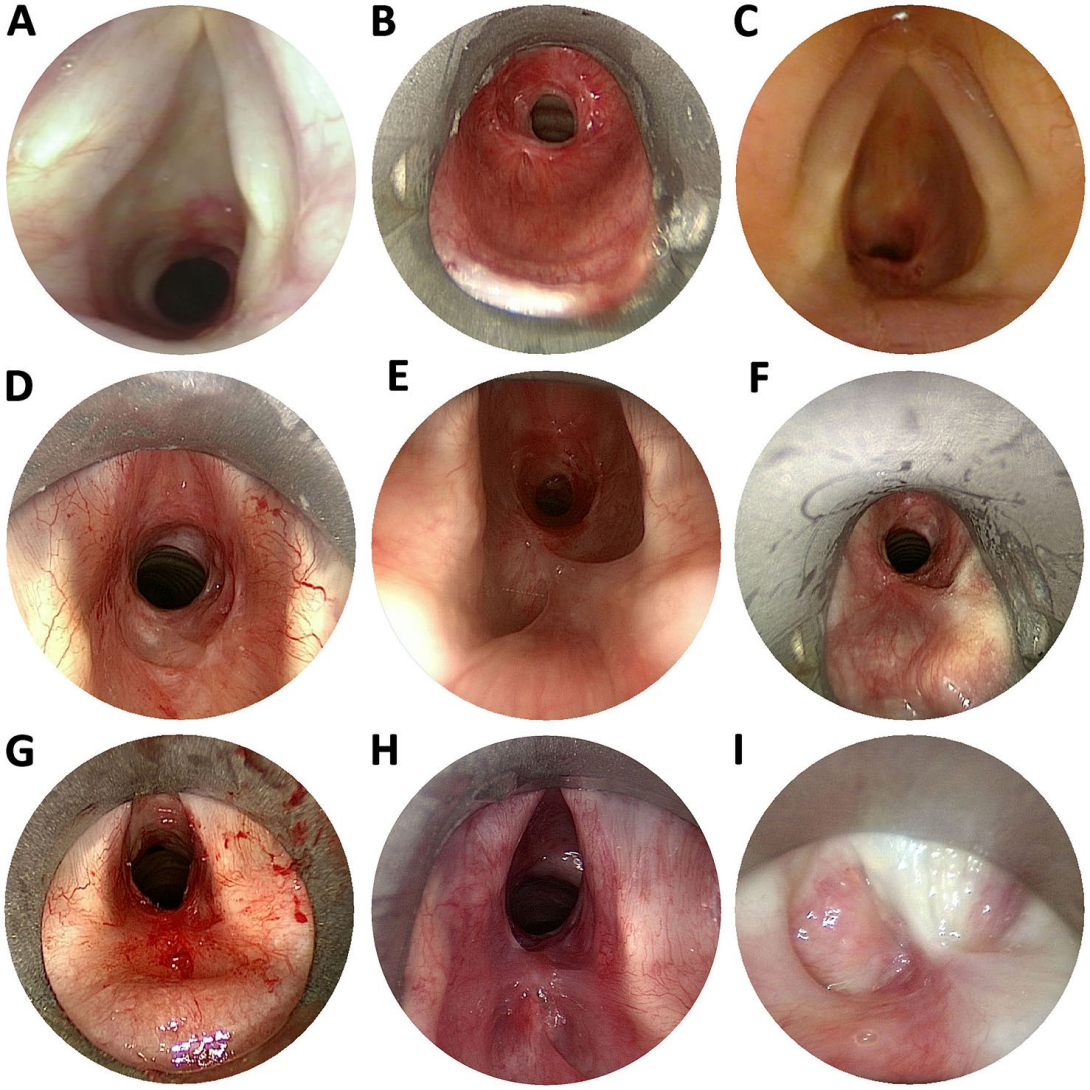
Presumed evolution of proximal stenosis progression in idiopathic subglottic stenosis, obtained from a cross-sectional survey of different patients with the condition at different stages. **a** A patient with an early and non-obstructive recurrence of ISS. Both fibrotic scar and the acute inflammatory process which will lead to stenosis reformation are evident; **b**, **c**. Two patients with ISS who show true subglottic stenoses, which is closer to, but still separate from the inter-arytenoid area in the case of **c** compared with **b**. **d** An early recurrent stenosis showing areas of abnormality within the inter-arytenoid area. **e**, **f**. Two patients with recurrent ISS showing early scar formation involving the posterior glottis. Both patients had normal vocal fold mobility. **g**, **h** Two patients with more advanced inter-arytenoid scarring. The scars impaired but did not completely prevent vocal mobility. **i** Total glottic obliteration caused by aggressive laser treatment of idiopathic subglottic stenosis

We have noted that in some patients who undergo serial endoscopic treatments, over the course of successive interventions, that the stenosis may progress proximally to involve the immediate infraglottic and glottic areas (Fig. 1). Glottic involvement in ISS shortens symptom-free interval following subsequent endoscopic treatments and reduces the likelihood of success of open surgery [4]. In extreme cases, proximal stenosis progression can cause complete obliteration of the laryngeal lumen (Fig. 1), the subsequent treatment of which may then require a laryngectomy [4]. The aim of the current study was to report the incidence of, risk factors for, and consequences of vocal fold fixation due to proximal stenosis progression in patients with ISS in a tertiary airway unit.

## Patients and methods

### General

Patients treated for ISS were identified by reviewing a database of all laryngological surgical procedures undertaken in the department from January 2004 to December 2016. A chart review was undertaken to record information about patient age at first treatment, comorbidities, body mass index, and presence of gastro-oesophageal reflux requiring regular medication. The diagnosis of ISS was based on the patient’s most recent diagnosis. Patients with this condition undergo regular review for development of other symptoms that may suggest a systemic vasculitis and appropriate blood tests are performed as indicated.

### Details of treatments

**Fig. 2:**
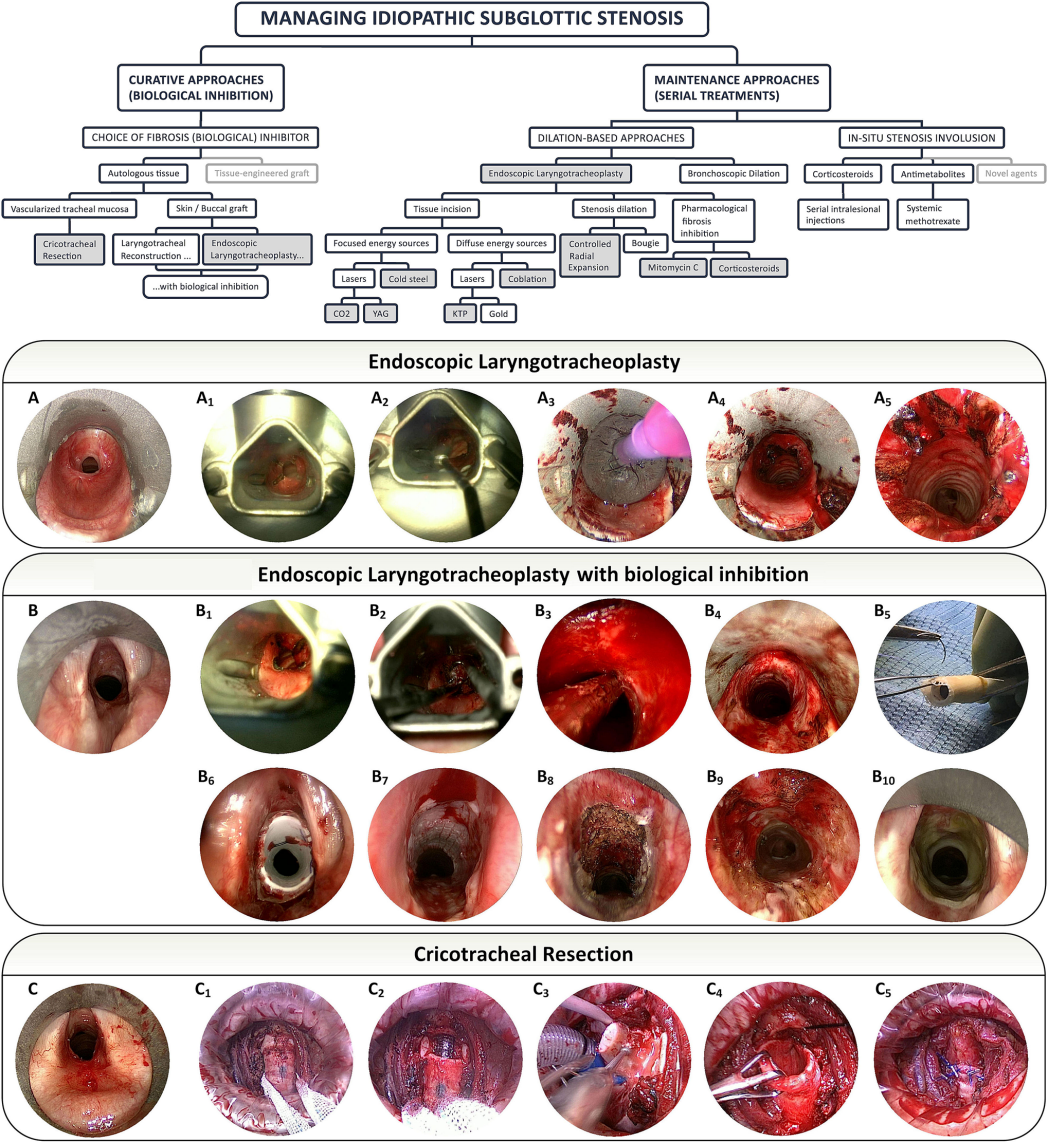
A treatment approach for managing idiopathic subglottic stenosis. Treatment approaches used in the present study are highlighted in grey. Boxes highlighted in grey represent treatments used in the present series. Technical description of endoscopic laryngotracheoplasty: **A** preoperative endoscopies appearances of an ISS patient undergoing a standard endoscopic laryngotracheoplasty. Access is achieved using suspension laryngoscopy with a Dedo-Pilling surgical laryngoscope. **A1** The first step in this procedure is cruciate incision of the stenosis, which may be performed with cold steel, a CO_2_, YAG or KTP laser, or coblation. **A2** Intralesional injection of 60–80 mg of triamcinolone acetonide (Kenalog^®^; Bristol-Myers Squibb Co; New York, USA) into and around the lesion. Steps **A1** and **A2** may be performed in reverse order where the severity of the stenosis does not raise concern of causing near-total obstruction in the time between steroid injection and stenosis incision. **A2**, **A3** The stenosis is then dilated using a controlled radial expansion balloon (CRE™, Boston Scientific Corp, Fremont, USA) Most patients are dilated to 16.5 mm, but patients with larger intrinsic subglottic diameter may be dilated to 18 mm. Dilation is typically applied for two cycles of 90 s and care is exercised to avoid applying shearing forces on the stenosis due to balloon slippage. Furthermore, the shortest available balloon length should be used to minimise application of radial forces to normal proxomal or distal mucosa which, in ISS, has a significant to fibrosis. **A4**, **A5** The immediate post-dilation result. Note the presence of intact mucosal bridges between the incisions. Technical description of endoscopic laryngotracheoplasty with biological inhibition performed according to its original description [1]. **B** Preoperative appearance of an ISS patient undergoing endoscopic laryngotracheoplasty with biological inhibition. **B1** Suspension laryngoscopy is established using the Dedo-Pilling laryngoscope and cruciate laser incisions are made as per **A1**. The purpose of these incisions are to provide a depth gauge for the next stage and to provide areas over which a skin graft is less likely to ‘take’, in order to encourage later respiratory remucosalisation. **B2** Cold-steel debulking excision of the stenosis. **B3** Circumferential excision of the stenosis using a 27 cm tricut-blade tracheal microdebrider (Medtronic, Minneapolis, USA). Particular care is exercised, especially when treating the posterior aspect of the stenosis and most particularly when the stenosis extends to the proximal cervical trachea, to avoid creating a tracheo-oesophageal fistula. For this reason, the surgery is only performed when there is recurrence of the stenosis and good judgement of stenosis depth may be obtained from the laser cuts. **B4** Endoscopic views following circumferential subglottic and/or cervical tracheal mucosectomy and lamina propria removal. **B5** Construction of a stent from the cut limb of a 12 mm thoracic Montgomery T-tube (Boston Medical Products Inc, Shrewsbury, USA). The stent is sized to cover a distance from the conus elasticus [25] to 1.5 cm below the lower border of the mucosectomy. The stent is covered with a paraffin sheet and a 0.01″ split-thickness skin graft. The cut dermal side points outwards and the skin/paraffin sheet is sutured to the silicone stent using a single 4/0 monofilament suture. The stent is secured with a single 0 nylon stent-holding suture that is placed through the lateral tracheal wall, the stent (**B6**), and is brought out through the opposite tracheal wall and is sutured subcutaneously. More precise technical descriptions of how to construct a stent-holding suture [29] and how to suture the paraffin sheet/skin graft composite to the silastic stent [4] have been reported previously. By cutting a Montgomery T-tube, the tube will contain a smooth native end and a rough freshly-cut end. The smooth end is placed proximally. The patient stays in hospital for 2 weeks and receives humidification and 5 days of postoperative antibiotic prophylaxis. No tracheotomy is placed and the patient resumes eating and drinking on the first postoperative day after speech and language therapy assessment. **B7** At 2 weeks, the stent-holding suture is cut endoscopically and the stent is removed. All skin is removed using suctioning at this point. The patient is discharged the next day. **B8** Three weeks later, the patient undergoes an endoscopic procedure where two of four quadrants of skin is lasered. **B9** Three weeks after that, a further mucosal resurfacing is performed on the two other quadrants and this treatment marks the end of the planned interventions. Any intervention after this point for any indication represents a treatment failure. **B10** Treatment failure secondary to airway crusting secondary to persistent keratinisation of the airway despite adequate resurfacing. Technical description of cricotracheal resection (CTR): **C** the immediate preoperative view of an ISS lesion, which had extended proximally and involved the immediate infraglottic area. **C1** The proximal trachea is mobilized through a standard cervical collar incision, taking care to avoid trauma to the recurrent laryngeal nerves. To achieve this, dissection is performed directly onto the tracheal surface and recurrent laryngeal nerves are not formally identified. **C2** In this patient a voice-sparing cricotracheal resection was not performed and the anterior arch of the cricoid cartilage was resected to allow access to the subglottis and to remove disease-bearing tissue. **C3** The diseased subglottic mucosa is resected and the remaining lateral and posterior elements of the cricoid cartilage are thinned with a drill both to remove pathological tissues and to create space into which the trachea is pulled up. **C4** The mobilised trachea is brought superiorly to sit within the subglottis. The posterior wall of the trachea is sutured to the posterior glottic mucosa using PDS. **C5** The anterior tracheal wall being sutured to the inferior border of the thyroid cartilage using Prolene, thus completing the anastomosis. Postoperatively the patient is transferred to the ITU intubated, and is brought back to the operating room 48 h later for extubation and laryngotrachesocpy. A soft collar is worn in reverse by the patient to discourage neck extension for 6 weeks

Details of endoscopic and open treatments (Fig. 2) were recorded. Laser or cold-steel incisions were used over the course of the series depending on the availability and surgeon preference. Coblation (Smith & Nephew, London, UK) and potassium titanyl phosphate (KTP) laser, which diffusely impart energy to the tissues, were also used at different times over the course of the series depending on availability and surgeon preference. The method of tissue incision was divided into focused energy and diffuse energy (Fig. 2) [19], and this was used as a stratification variable. Patients had steroid injections and/or mitomycin C applications at different points during the course of their treatments depending on availability and surgeon preference. Glottis-to-stenosis (GtS) distance was measured from the medial edge of the vocal folds to the proximal level of the stenosis, and proximal stenosis progression was defined as a reduction in this distance over the course of successive treatments. Vocal fold fixation due to proximal stenosis progression was defined as the occurrence of significant voice or airway complications in the context of glottic involvement and was calculated as an actuarial variable. Functional outcome was recorded at the last follow-up using a disease-specific patient-reported outcome instrument measuring the domains of dyspnoea, voice, swallowing, cough/mucus, and independence, principally from the need to use regular humidity to maintain the airway [20].

### Compliance with ethical standards

Ethical approval for the study was granted by the Auckland Hospital Research Office. Specific consent for publication was not sought from individual patients. None of the authors have any conflicts of interest arising from the study and the study did not receive funding from any sources.

### Data analysis

Continuous and categorical variables were expressed as means with standard deviations and proportions, respectively. A timeline plot was constructed to illustrate the number and nature of endoscopic and open airway treatments, as well as occurrence and consequences of vocal fold fixation. Log-rank statistics was used to examine the association between different variables and occurrence of proximal stenosis progression. Cox regression was used to identify independent predictor(s) of vocal fold fixation due to proximal stenosis progression. Analysis was performed using MedCalc (MedCalc Software bvba, Acacialaan, Belgium) and *p* < 0.05 was considered significant.

## Results

### General

Between 2004 and 2016, 22 patients with ISS were treated. All patients were female and the mean age at first treatment was 46 ± 7 years (range 31–58). Mean body mass index was 30 ± 9 kg m^−2^ (range 20–55). Fourteen patients (64%) were receiving regular medications for gastro-oesophageal reflux. At the time of first treatment, the mean GtS distance was 18 ± 6 mm (range 5–27) and five patients had GtS distances < 15 mm at initial presentation.

### Treatment details

**Fig. 3:**
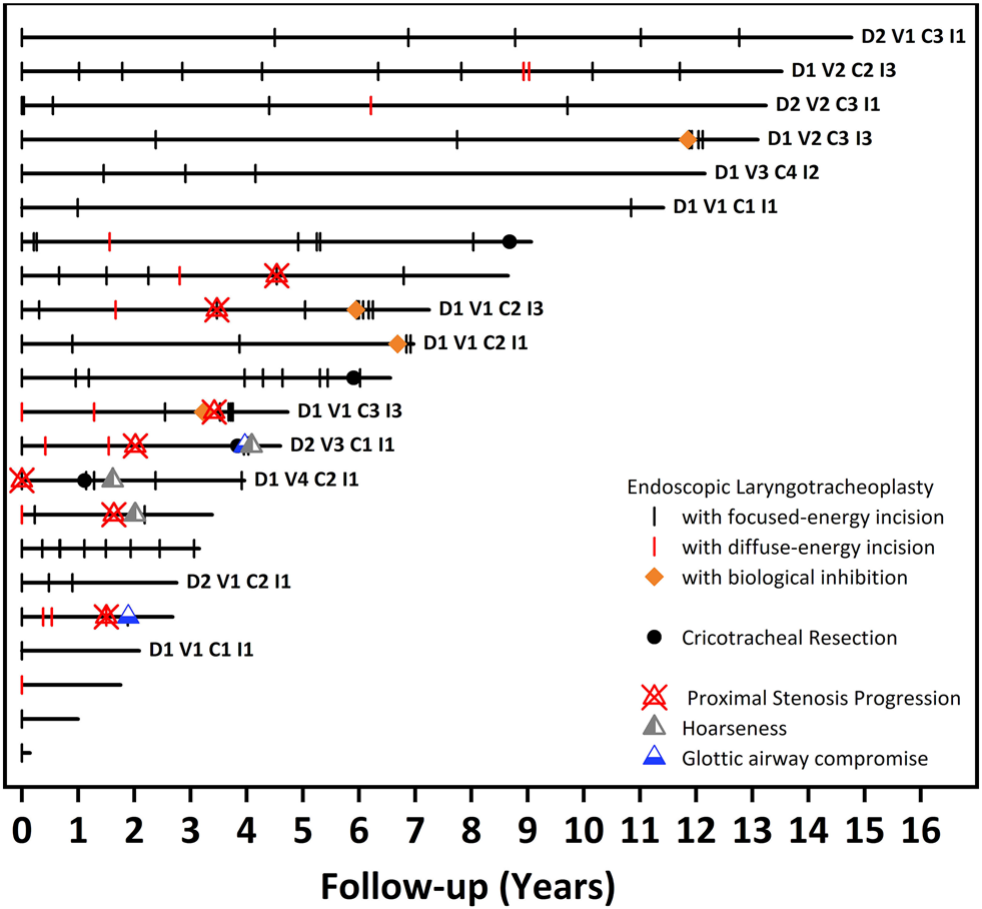
Timeline plot of patients with ISS. All patients had A1 (prosthesis-free) and S1 (normal swallowing) status. Dyspnoea (D), voice (V), cough (C), and independence from use of humidity (I) are presented

All patients received laser surgery using primarily CO_2_ laser at least one time over the course of their treatment. All patients had either intralesional steroids and/or topical mitomycin C during at least one time over the course of their treatments. Nine patients (41%) had coblation treatment at least once. Four patients had an endoscopic laryngotracheoplasty with biological inhibition as previously described [1] and four patients had a cricotracheal resection (Fig. 2). Figure 3 provides patient-level timelines of treatments, follow-up, and functional outcome.

### Vocal fixation due to proximal stenosis progression

**Fig. 4:**
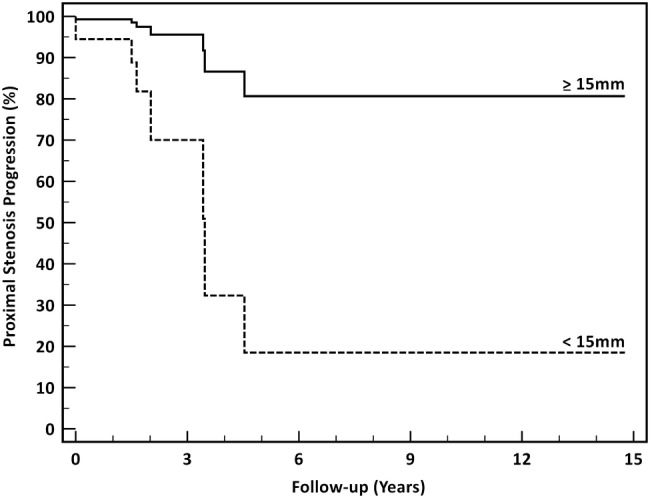
Adjusted probability of proximal stenosis progression as a function of glottis-to-stenosis distance at the time of first treatment. The inset shows the Cox regression model which identifies glottis-to-stenosis distance as the only independent predictor of proximal stenosis progression, with the use of diffuse energy incision devices (coblation and KTP laser) approaching statistical significance on multivariable analysis

Vocal fold fixation due to PSP occurred in seven patients (32%). In three patients, this complication led to an increase in the frequency of treatments to treat inter-arytenoid scarring, but to date no permanent consequences have been observed in those patients. In two patients, unilateral vocal fold fixation led to permanent rough and breathy dysphonia. Both patients had pre-treatment unilateral vocal fold mobility impairment and needed to have high cricotracheal resections. Two further patients had glottic airway compromise due to bilateral vocal fold mobility impairment. In one of these two patients, glottic airway compromise necessitated emergency admission to intensive care following a life-threatening episode, and in a second patient, it led to chronic glottic airway insufficiency. No airway-related deaths occurred and no patient required a tracheostomy. Table 1 provides univariable and multivariable analyses of variables associated with proximal stenosis progression. The only independent predictor of vocal fold fixation due to proximal stenosis progression was a GtS distance of < 15 mm at the first treatment (Fig. 4).

**Table 1:** Variables associated with vocal fold fixation due to proximal stenosis migration

	Univariable (Log-rank) analysis			Multivariable (Cox regression) analysis		
	HR	95% CI	*p*	Exp(*b*)	95% CI	*p*
Age > 50 years	0.55	0.10–3.05	0.49	0.47	0.03–7.97	0.60
BMI > 35 kg m^−2^	0.84	0.17–4.11	0.83	0.51	0.08–3.30	0.48
Diffuse energy (coblation and KTP laser) incision	4.83	1.09–21.43	**0.04**	22.05	0.76–642	0.07
> 4 dilations	0.58	0.11–2.96	0.51	0.40	0.06–2.71	0.35
Initial glottis-to-stenosis distance < 15 mm	7.77	1.01–60.23	**0.04**	7.85	1.15–53.55	**0.04**
Gastro-oesophageal reflux disease	2.41	0.50–11.58	0.27	0.60	0.02–15.40	0.76

Bold values are statistically significant

*HR* hazard ratio, *95% CI* 95% confidence interval

## Discussion

In the current study, vocal fold fixation due to proximal stenosis progression occured in a significant proportion of patients with ISS who were treated with serial endoscopic treatments, particularly when cruciate stenosis incision was performed using diffuse energy sources. The occurrence of vocal fixation due to proximal stenosis progression led to permanent voice and airway complications. Glottic involvement in ISS is known to worsen prognosis, both by reducing the efficacy of subsequent endoscopic treatments and the success and safety of both resection and reconstruction-based airway treatments [1]. The only independent risk factor for this complication in the present series was a short distance (< 15 mm) between the glottis and the proximal stenosis at the time of first treatment. However, it is important to recognise that all stenoses that progressed to involve and compromise glottic function started as discrete lesions of the subglottis and/or cervical trachea. There was a significant association on univariable analysis between the use of diffuse energy sources and increased risk of vocal fixation, but this did not remain significant on multivariable analysis. Coblation and KTP laser are known to impart energy more diffusely to the tissues and to ablate a larger surface area of tissue, with depths of penetration measured in millimetres, as opposed to incisions achieved either by CO_2_ laser [19] or cold steel. A multicentre collaborative study is needed to better elucidate the relationship between the method of tissue incision, especially with respect to the amount of energy and the severity of trauma imparted to the tissues and long-term outcome. We found no relationship between the number of dilations and vocal fold fixation and, indeed, patients in this and other series underwent large number of endoscopies [21].

At present, the principal consideration of whether or not to recommend continuation of serial endoscopic treatment to those patients who prefer it is the symptom-free interval. It is known that glottic involvement [4] and the need to perform more extensive cricotracheal resections [22] reduce both symptom-free interval and increase the likelihood of recurrence. More importantly, they diminish the efficacy and safety of definitive treatments which could have been used more safely and to better effect earlier in the natural history of the patient’s disease. It is recommended therefore that stenosis configuration and GtS distance should be assessed and compared to previous appearances during every patient visit. An evolving proximal stenosis progression should be identified and treated before it can lead to vocal fold fixation. Definitive treatment should be offered while a sufficient length of healthy infraglottic mucosa remains to accommodate the anastomosis fashioned during a cricotracheal resection [23, 24], or full coverage of the proximal extent of the disease by a skin graft during an open laryngotracheal reconstruction [4] or an endoscopic laryngotracheoplasty with biological inhibition [1] procedure. Further investigation is required to determine whether, at the time that proximal stenosis progression is observed initially, but before it has caused vocal fold fixation, the proximal stenosis progression can be halted with pharmacological agents such as methotrexate [15], mitomycin C [11, 26], or intralesional steroids [13, 14, 27], or whether a definitive procedure should be performed expeditiously.

In conclusion, vocal fold fixation due to proximal stenosis progression is a major complication, likely of serial endoscopic treatment, of ISS. The permanent sequelae of proximal stenosis progression, principally hoarseness, are the very complications patients seek to avoid by opting for endoscopic treatment over definitive procedures. Proximal stenosis progression can be acutely life threatening and could worsen the long-term efficacy of subsequent definitive airway surgery. Patients who initially present with lesions that are close to the glottis are at higher risk of this complication. The use of diffuse energy tissue incision methods may put the patient at more risk of developing proximal stenosis progression. Therefore, it would be prudent to minimise the amount of energy and trauma that is imparted to the tissues at every endoscopic treatment for ISS. As single-modality disease-modifying treatments, like intralesional corticosteroid injections or systemic therapies, begin to be used and their efficacy [13, 14, 27] and safety profiles [28] become better understood, the role of serial endoscopic dilation may diminish, although whether or not proximal stenosis progression may occur with these novel treatments too, should be carefully studied. Stenosis configuration and location should be carefully assessed at every patient visit and compared with previous appearances to identify proximal migration of the stenosis before it can compromise efficacy of definitive treatment approaches, or cause actual vocal fold fixation.
